# Respiratory syncytial virus-neutralizing serum antibody titers in infants following palivizumab prophylaxis with an abbreviated dosing regimen

**DOI:** 10.1371/journal.pone.0176152

**Published:** 2017-04-24

**Authors:** Jennifer Claydon, Amitava Sur, Allison Callejas, Mihoko Ladd, Eddie Kwan, Richard Taylor, Stuart E. Turvey, Alfonso Solimano, Pascal M. Lavoie, Nico Marr

**Affiliations:** 1 Department of Pediatrics, University of British Columbia, Vancouver, Canada; 2 BC Children’s Hospital Research Institute, Vancouver, Canada; 3 Children's & Women's Health Centre of British Columbia, Vancouver, Canada; 4 Department of Pediatrics, University of Victoria, Victoria, Canada; 5 Canadian Center for Vaccinology, Halifax, Canada; St. Jude Children's Research Hospital, UNITED STATES

## Abstract

**Background:**

Monthly injections of palivizumab during the respiratory syncytial virus (RSV) season in at-risk infants reduces RSV-associated hospitalizations. However, the additive effect of naturally acquired immunity remains unclear. The objective of this study was to assess total neutralizing serum antibodies (NAb) against RSV in at-risk infants who had received an abbreviated course of palivizumab prophylaxis.

**Methods:**

Serum samples were collected from infants enrolled in the RSV Immunoprophylaxis Program in British Columbia, Canada over 2 consecutive RSV seasons (2013 to 2015). Infants in this program had received an abbreviated course of palivizumab in accordance with the provincial guidelines. Data were compared to adults and infants less than 12 months of age who did not receive palivizumab. Anti-RSV NAb titers were measured using an RSV microneutralization assay.

**Findings:**

Infants who received palivizumab had anti-RSV NAb titers at the end of the RSV season that persisted beyond what is expected from the pharmacokinetics of palivizumab alone. Moreover, 54% of the control infants who did not receive palivizumab and all tested adults had protective anti-RSV NAb titers.

**Conclusions:**

Based on our observations, we hypothesize that naturally acquired NAb provide additive protection, which may significantly reduce the need for additional doses of palivizumab in infants at risk of severe RSV infections.

## Introduction

Respiratory syncytial virus (RSV) is the main cause of lower respiratory tract infections and hospitalization among infants and young children, and is responsible for up to 200,000 fatalities in these age groups each year, worldwide [1]. Two randomized, double-blinded, prospective placebo-controlled trials [2, 3] have shown that 5 monthly intramuscular injections of palivizumab reduce the risk of hospitalization by about half in infants born prematurely below 36 weeks gestational age (GA) with and without bronchopulmonary dysplasia] and in children with hemodynamically significant congenital heart disease [3]. Palivizumab is a humanized monoclonal anti-RSV neutralizing antibody given at 15 mg/kg body weight during each injection [2, 3]. Based on the available safety and efficacy data, many medical jurisdictions in high-resource countries have introduced palivizumab prophylaxis programs for high risk infants adopting the 5-dose-regimen as used in the clinical trials. We recently reported hospitalization rates among at–risk infants in British Columbia (BC), Canada, who received an abbreviated palivizumab regimen of 3 or 4 doses during an RSV season that were comparable to historical controls treated under a 5-dose regimen [4]. It remained unclear whether natural anti-RSV neutralizing antibodies (NAb) contributed to the protection of these infants who received an abbreviated palivizumab dosing schedule. Preterm infants, with the exception of those born ≤28 weeks GA, have serum levels of maternal RSV F protein-specific serum IgG at birth that are comparable to that of term infants [5]. Moreover, it has been shown that even young infants are capable of producing anti-RSV NAb following RSV infection, and that preexisting maternally derived antibodies in young infants, rather than age, is the most important factor influencing this response [6]. Previous studies have also demonstrated associations between the seasonal variation of maternally derived anti-RSV NAb and the seasonal pattern of RSV hospitalizations in infants at the population level [7], as well as between breast feeding and lower risk for RSV hospitalizations in a case-control cohort [8]. These observations further indicate that maternally-derived antibodies contribute to the protection of infants during the course of primary RSV infection. An observational study of children with underlying heart or lung disease conducted by The Pediatric Investigators Collaborative Network on Infections in Canada (PICNIC) before the introduction of intramuscular palivizumab and intravenous polyclonal immune globulin (RSV-IGIV) prophylaxis had demonstrated an U-shaped distribution of serum anti-RSV NAb levels with increasing age [9], further indicating that natural humoral immunity against RSV is acquired both passively and actively in early life. Here we report accumulative serology data from infants in the British Columbia Immunoprophylaxis Program who had received an abbreviated course of palivizumab prophylaxis [4]. We observed protective anti-RSV NAb titers up to day 105 after the last dose of palivizumab and hypothesize that in these infants, prolonged protection is provided through naturally acquired antibodies due to subclinical or mild RSV infection.

## Materials and methods

### Sample collection

All infants who were approved to receive palivizumab in accordance with the BC Immunoprophylaxis Program guidelines [4] were eligible to participate in our study. Approved infants were enrolled at the Children’s & Women’s Health Centre (Vancouver, Canada) during the 2013/14 and 2014/15 seasons or at the Victoria General Hospital (Victoria, Canada) during the 2014/15 season and the analysis was carried out on an intention-to-treat basis. Serum samples were collected at the end of the RSV season (at least 28 days after the last palivizumab dose or after April 15, whichever came last). Our goal was to collect serum samples as soon as possible after the set criteria but no end date was set for serum collection. To maximize the sample size, we accepted all children into the study for whom written consent was obtained, even if the serum sampling was delayed due to unavailability of the parents/children. In addition, we recorded gender, reason for referral in accordance with the BC Immunoprophylaxis Program guidelines, age (in months) at serum collection, number of days between the last dose and serum collection as well as number of doses administered and intervals between the doses. The latter information was collected to verify that all infants who were enrolled into our study had successfully completed the recommended dosing regimen. For comparison, de-identified blood samples were obtained from two control groups of healthy adults (at the end of both 2013/14 and 2014/15 RSV seasons) and infants ≤12 months of age (at the end of the 2013/14 RSV season only) who did not receive any palivizumab. More specifically, samples of adult subjects were from volunteers aged 19–50 who had been enrolled in other unrelated studies as healthy adult control subjects for which recruitment took place at Children’s & Women’s Health Centre or its affiliated BC Children’s Hospital Research Institute (Vancouver, Canada). Samples of the infant control group were surplus serum from patients who had been admitted to (inpatients) or visited (outpatients) the Children’s & Women’s Health Centre (Vancouver, Canada) during the 2013/14 RSV season and for which serum collection had been requested by the treating physician for any diagnostic purpose. No inclusion and exclusion criteria other than age and ineligibility for palivizumab prophylaxis were applied for this group. Blood was collected in microtainer tubes without additives (Becton Dickenson), incubated at room temperature for 30 to 120 min, followed by 10 min centrifugation at 1000 *g* to separate serum from coagulate, and stored at -80°C for batch analyses. Prior to sample and data collection, we obtained informed written parental consent on behalf of all participating children who were approved to receive palivizumab. Serum samples from control subjects collected for this study were completely deidentified leftover specimen and data were analyzed anonymously. The study was approved by the University of British Columbia / Children’s and Women’s Health Centre of British Columbia Research Ethics Board.

### RSV microneutralization assay

Anti-RSV NAb were quantified using a modification of a microneutralization assay as described by Krajden *et al*. [10]. Serial 2-fold dilutions of heat-inactivated sera (30 min, 56°C) were prepared in serum-free Minimum Essential Medium (MEM) supplemented with 1x non-essential amino acids (NEAA), 2 mM L-glutamine, and 1 mM sodium pyruvate (HyClone), starting at 1:4. Serial serum dilutions were incubated at 37°C for 60 min with an equal volume of the recombinant GFP-expressing strain rgRSV30 [11] to provide ~100 pfu per 50 uL. 50 uL of the serum-virus mixtures were added in duplicate to monolayers of HEp-2 cells in 96-well flat-bottom tissue culture-treated microtiter plates, and incubated at 37°C with 5% CO_2_. Two hours post-infection, the inoculum was replaced with fresh MEM with NEAA, 2 mM L-glutamine, 1 mM sodium pyruvate, Pen/Strep, 10% heat-inactivated fetal bovine serum (HyClone), and 1% agarose, which was adjusted to 42°C prior to addition to the infected HEp-2 cells. After 2 to 4 days of virus propagation at 37°C and 5% CO_2_, GFP-stained syncytia were counted using an inverted fluorescence microscope (Nikon Eclipse TS100-F). Endpoint of the assay was the neutralizing titer (NT_95_), designated by the highest serum dilution in the well where ≥95% reduction of the initial infection dose was observed. Final titers were determined by calculating the mean of the specific dilution of each serum sample tested in duplicate times two, since each serum dilution was mixed with an equal volume of the challenge virus, rgRSV30. Serial two-fold dilutions of palivizumab (Abbott), beginning either at 100 μg/mL, or 40 μg/mL, and virus-free MEM were used as controls. In addition, a virus back titration was performed for each experiment, to validate the initial infection dose. As done in other studies [12–14], we considered a palivizumab serum concentration of greater or equal to 40 μg/mL to be protective. This value has been associated with a reduction of pulmonary RSV replication by more than 100-fold in the cotton rat model [15]. Thus, in our assay we defined the median NT_95_ equivalent to 40 μg/mL of palivizumab as our minimum protective threshold.

### Statistical analysis

Data were analyzed by standard descriptive methods using the GraphPad Prism. Normal distribution within each group was assessed using the D'Agostino & Pearson normality test. Kruskal-Wallis and Dunn’s post-tests or Mann Whitney tests were used to compare NAb levels between groups. We performed Wilcoxon Signed Rank Tests to compare the median anti-RSV NAb level of each group against a hypothetical value that we had defined as the minimum protective threshold in this study. The relationship between anti-RSV NAb levels and time since final dose or age in infants who had received palivizumab prophylaxis was analyzed using Spearman correlation analysis. *P*< 0.05 was considered statistically significant.

## Results

**Fig 1:**
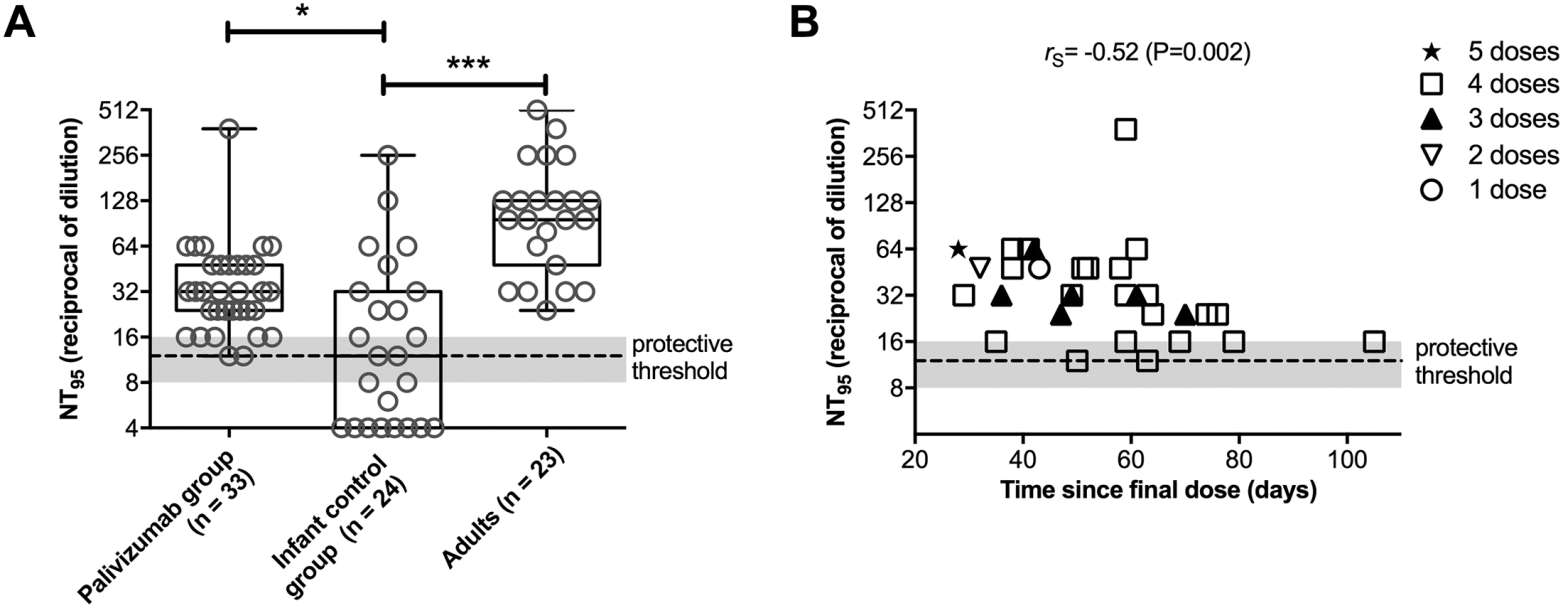
End-of-season anti-RSV NAb levels in at-risk infants who had received palivizumab prophylaxis in accordance with the BC RSV immunoprophylaxis program guidelines that were in effect during the 2013/14 and 2014/15 seasons (palivizumab group) and in control subjects. **A,** Comparison of anti-RSV NAb levels (expressed as neutralizing titers (NT_95_)) between infants of the palivizumab group and two control groups of infants ≤12 months of age and healthy adults who did not received palivizumab. **B,** Anti-RSV NAb levels in the palivizumab group plotted against the number of days since they had received the final dose of palivizumab. Dashed lines indicate the median NT_95_ (grey shaded area indicates min and max value) equivalent to 40 μg/ml palivizumab (defined as the minimum protective threshold). ***, P < 0.001; ****, P < 0.0001; r_S,_ Spearman correlation coefficient.

Anti-RSV NAb levels were determined in infants who had received palivizumab in accordance with the BC RSV Immunoprophylaxis Program guidelines that were in effect during the 2013/14 and 2014/15 seasons [4]. The demographic and clinical characteristics of the participating infants are shown in Table 1. All participating infants had successfully completed the palivizumab prophylaxis regimen in accordance with the BC RSV Immunoprophylaxis Program guidelines. One of the infants had received a 5^th^ post-operative dose following cardiac surgery and prior to serum collection. Healthy adults and pediatric patients ≤12 months of age who did not receive any palivizumab served as controls. In the serum of infants who had received palivizumab, we detected anti-RSV NAb (median NT_95_: 32; min-max: 12–384) at or above levels equivalent to that of 40 μg/ml palivizumab, which was defined as the minimum protective threshold (median NT_95_: 12; min-max: 8–16) (Fig 1A). The average duration between final dose of palivizumab and the time of serum sample collection was 55 days (min-max: 28–105). In the control infant group, only 13 (54%) out of 24 individuals had protective antibody titers (median NT_95_: 12; min-max: ≤4–256), whereas all tested adults had protective antibody titers (median NT_95_: 96; min-max: 24–512) (Fig 1A). NT_95_ values in neither of the groups were normally distributed and Kruskal-Wallis one-way analysis of variance showed significant variation of the anti-RSV NAb levels between all three groups tested (P<0.0001). When we compared the mean rank of the palivizumab group and adults with that of the infant control group using Dunn's multiple comparisons post-test, we found that both the palivizumab group and the adult controls were significantly different from the control group of infants ≤12 months of age who did not receive palivizumab (Fig 1A). Furthermore, we used Wilcoxon Signed Rank Tests to compare the median anti-RSV NAb level of each group against a hypothetical value set at 12—the median NT_95_ value that we had defined as the minimum protective threshold—and found the palivizumab group and the adult group to be significantly different from the set hypothetical median (P < 0.0001), whereas the median of the infant control group was not. Notably, even children who had received only 3 doses of palivizumab had anti-RSV NAb levels above the minimum protective threshold up to 70 days after they had received their final dose (Fig 1B), which differed greatly from the projected duration of protection based on pharmacokinetic models of palivizumab alone [12, 16]. We observed a negative correlation between the anti-RSV NAb levels among infants who had received palivizumab and time since their final dose (r_s_ = -0.52, P = 0.002) but were unable to generate a robust regression model that would allow us to approximate the half-life and duration for the interception with the minimum protective threshold (R^2^ ≤ 0.274, data not shown). No significant differences in the NT_95_ values were found when stratifying the palivizumab group in two subgroups of children that had received ≤3 doses versus children that had received ≥4 doses (Mann Whitney test, data not shown). There was also no significant correlation between the age at sample collection in the palivizumab group and the measured NT_95_ values (Spearman correlation, data not shown).

**Table 1:** Clinical and demographic characteristics of infants who received palivizumab versus control infants.

Characteristics	Palivizumab group	Control group
**Median age in months** (25% percentile; 75% percentile)	8 (5.5; 15)	6.5 (4.25; 11)
**Gender**		
• Male	19	13
• Female	14	11
**RSV season**		
• 2013–14	17	24
• 2014–15	16	0
**Number of doses received**		
• 1	1	NA
• 2	1	NA
• 3	7	NA
• 4	23	NA
• 5	1*	NA
**Reason for referral**		
• BPD	6	NA
• GA <29 weeks	7	NA
• GA 29 to <35 weeks^#^	4	NA
• CHD	6	NA
• CF	5	NA
• Other	5	NA

Controls were infants ≤12 months of age who did not receive any palivizumab. NA, not applicable; BPD, bronchopulmonary dysplasia; GA, gestational age; CHD, congenital heart disease; CF, cystic fibrosis;

*, infant received a 5^th^ post-operative dose following cardiac surgery and prior to serum collection;

^#^, infants with a risk factor score ≥42 points. See Lavoie *et al*. [4] for details on the administration criteria and calculation of the risk factor score for palivizumab prophylaxis in British Columbia, Canada, during the 2013/14 and 2014/15 seasons.

## Discussion

Here we report that at-risk infants who received 3 or 4 monthly doses of palivizumab during an RSV season maintained anti-RSV NAb levels beyond what is expected based on the pharmacokinetics of the drug alone. These findings are in agreement with our recently published population-based outcome data demonstrating that adequate protection against RSV hospitalization can be achieved using an abbreviated palivizumab dosing schedule [4].

Based on the pharmacokinetics of palivizumab, some authors argued that administering less than 5 doses of palivizumab would be sub-optimal [12, 16]. We hypothesize that the discrepancy between this argument and our “real-life” experience is explained by the combined effect of palivizumab and naturally acquired anti-RSV NAb, which provide additive protection. To our knowledge, this is the first report of serological outcomes in infants who received palivizumab prophylaxis and takes into account both palivizumab-related and natural humoral protection. In support of our hypothesis, a considerable proportion (54%) of a control group of infants ≤12 month of age who did not receive palivizumab also had protective levels of anti-RSV NAb. To which extent this was due maternally acquired antibodies or primary RSV infection could not be discriminated with our assay. Our data show that levels of anti-RSV NAb were significantly higher among healthy adults in comparison to the control infant group who did not receive palivizumab, suggesting that repeated exposure to RSV has a positive effect on the development of humoral immunity against this virus.

Estimating the duration of protection for any dosing schedule remains challenging, in part because the commonly used minimum protective threshold of 40 μg/ml palivizumab has been defined rather arbitrarily based on animal studies [15]. In the clinical development of palivizumab, the goal has been to define an intramuscular dosing regimen to maintain trough concentrations of at least 25 to 30 μg/ml and ideally concentrations >40 μg/ml [17].

Our results suggest that natural anti-RSV NAb may rapidly provide additional protection beyond the first few months of age and this does not appear to be majorly impaired in at-risk infants who are also receiving monthly palivizumab injections. The development of naturally acquired humoral immune protection—as we show here even in high-risk children and in the absence of clinically significant infection—may explain, at least in part, why hospitalization rates following an abbreviated palivizumab regimen as used in British Columbia were comparable to hospitalization rates following a 5-dose-regimen [4]. Further studies are required to support this contention. There are a number of reasons why an abbreviated palivizumab dosing schedule may be just as effective as a five dose schedule, including (i) the maturation of immunity (including cell-mediated immunity) and ongoing post-natal lung development with increasing age; (ii) passive humoral immunity conveyed by the accumulation of antibody through maternal transfer during the 3^rd^ trimester of pregnancy, breast feeding, and/or with each additional dose of palivizumab; as well as (iii) the development of active immunity following (subclinical) primary RSV infection. Our findings also raise the question whether palivizumab prophylaxis can safely be discontinued in infants who experienced primary RSV infection regardless of the clinical presentation, like in those who experienced breakthrough RSV hospitalization [18]. This needs further investigation.

It is also important to highlight limitations of our study. We conducted an observational study in which we have assessed anti-RSV NAb titers in a relatively small number children in the British Columbia Immunoprophylaxis Program who had completed an abbreviated course of palivizumab prophylaxis in accordance with the provincial guidelines. The sample size is neither representative for the entire at-risk population to draw conclusive recommendations for palivizumab prophylaxis, nor has this study been designed to assess the kinetics of natural anti-RSV NAb and palivizumab. There is presently no proven method for measuring the neutralising activity of natural anti-RSV NAb and palivizumab independently of each other. Moreover, the anti-RSV NAb titer that we and others [12–14] have defined as the minimum protective threshold is based on studies in animal models [15, 17]. No human studies have validated whether this threshold is adequately defined.

In summary, our observations suggest that naturally acquired NAb may provide additive protection against RSV in children who are eligible for palivizumab prophylaxis. Our data also suggest a potential mechanism supporting the use of an abbreviated course of palivizumab prophylaxis in infants at risk of severe RSV infections, which appears to have a similar therapeutic benefit as the 5-dose-schedule prescribed by the manufacturer [4]. This hypothesis requires rigorous testing in future studies using adequate study designs and larger sample sizes.

## Supporting information

S1 Table Individual deidentified data. A, B and C: Subtables with individual deidentified data including NT_95_ values, relevant clinical and demographic characteristics of infants who received palivizumab (A) as well as of control groups of infants ≤12 months of age (B) and healthy adults (C) and who did not receive any palivizumab.
(XLS)
